# Northwestern University Schizophrenia Data and Software Tool (NUSDAST)

**DOI:** 10.3389/fninf.2013.00025

**Published:** 2013-11-07

**Authors:** Lei Wang, Alex Kogan, Derin Cobia, Kathryn Alpert, Anthony Kolasny, Michael I. Miller, Daniel Marcus

**Affiliations:** ^1^Department of Radiology, Northwestern University Feinberg School of MedicineChicago, IL, USA; ^2^Department of Psychiatry and Behavioral Sciences, Northwestern University Feinberg School of MedicineChicago, IL, USA; ^3^Department of Biomedical Engineering, Center for Imaging Science, Johns Hopkins UniversityBaltimore, MD, USA; ^4^Department of Radiology, Washington University School of MedicineSt. Louis, MO, USA; ^5^Department of Psychology, Washington University School of MedicineSt. Louis, MO, USA

**Keywords:** data sharing, neuroimaging, schizophrenia, computational anatomy, cognitive data, meta data

## Abstract

The schizophrenia research community has invested substantial resources on collecting, managing and sharing large neuroimaging datasets. As part of this effort, our group has collected high resolution magnetic resonance (MR) datasets from individuals with schizophrenia, their non-psychotic siblings, healthy controls and their siblings. This effort has resulted in a growing resource, the Northwestern University Schizophrenia Data and Software Tool (NUSDAST), an NIH-funded data sharing project to stimulate new research. This resource resides on XNAT Central, and it contains neuroimaging (MR scans, landmarks and surface maps for deep subcortical structures, and FreeSurfer cortical parcellation and measurement data), cognitive (cognitive domain scores for crystallized intelligence, working memory, episodic memory, and executive function), clinical (demographic, sibling relationship, SAPS and SANS psychopathology), and genetic (20 polymorphisms) data, collected from more than 450 subjects, most with 2-year longitudinal follow-up. A neuroimaging mapping, analysis and visualization software tool, CAWorks, is also part of this resource. Moreover, in making our existing neuroimaging data along with the associated meta-data and computational tools publically accessible, we have established a web-based information retrieval portal that allows the user to efficiently search the collection. This research-ready dataset meaningfully combines neuroimaging data with other relevant information, and it can be used to help facilitate advancing neuroimaging research. It is our hope that this effort will help to overcome some of the commonly recognized technical barriers in advancing neuroimaging research such as lack of local organization and standard descriptions.

## Introduction

Schizophrenia is a complex disease with heterogeneous clinical, behavioral, cognitive and genetic manifestations, and sharing of datasets is becoming essential in order to test hypotheses that can capture its variability and complexity (Poline et al., 2012). Case in point is the discovery of microRNA137 that succinctly illustrates the importance of data sharing: using computational biology techniques, Potkin et al. (2010) combined two previously published, separate datasets and discovered microRNA137 as a risk factor for schizophrenia. It should be noted that neither of the two distinct datasets had identified microRNA137. In a later confirmatory report on 51,695 individuals confirming microRNA137, the International Schizophrenia Consortium proclaimed that a new “cause” of schizophrenia had been found (Ripke et al., 2011).

In the neuroimaging front, the schizophrenia research community has witnessed consortium efforts such as the Functional Biomedical Informatics Research Network (FBIRN) (Friedman et al., 2008; Keator et al., 2008; Helmer et al., 2011), the Mind Clinical Imaging Consortium (MCIC) (Bockholt et al., 2010), and the Bipolar and Schizophrenia Network on Intermediate Phenotypes (BSNIP) (Tamminga et al., 2013), which have warehoused or federated increasingly large magnetic resonance imaging (MRI) datasets. These data sharing efforts have led to consistent findings of brain network dysfunctions within the dorsolateral prefrontal cortex (Potkin et al., 2009) and the rest of the cerebral cortex (Potkin and Ford, 2009) during memory-related tasks (Kim et al., 2009) and at rest (Allen et al., 2011). In a recent study (Turner et al., 2012) that combined structural MRI data from Northwestern University (described in this paper), FBIRN, and MCIC, we used a multivariate independent components analysis and found that two components, one including the medial temporal lobe, insula, inferior frontal and temporal lobes, and the other including the posterior occipital lobe, showed significant familiality.

In this paper, we describe the Northwestern University Schizophrenia Data and Software Tool (NUSDAST), an NIH-funded data sharing effort to stimulate new research. In making our existing neuroimaging data along with the associated meta-data and computational tools publically accessible, we have established a web-based information retrieval portal that allows the user to efficiently search the collection. Search and retrieval of relevant research information identification is an important part of the data sharing process that justifies the effort spent on data collection and storage and makes it useful to other scientists.

With 451 subjects, the majority of whom with longitudinal data, NUSDAST is one of the largest single-site, single-platform neuroimaging datasets related to schizophrenia, making it a uniquely important resource to share with the research community. NUSDAST will benefit the neuroscience community in many ways. First, scientists will be able to use these data to generate or test new hypotheses related to abnormalities of brain structures and neural networks in individuals with schizophrenia. Second, scientists will be able to rapidly replicate findings produced using their own datasets. Third, the data could be used to test and validate new brain mapping tools. Further, automatic image processing pipelines designed for the analysis of these datasets, which consists of neuroanatomical landmarking and diffeomorphic mapping tools along with training and validation datasets, will enable others to study other MR datasets collected from other clinical samples.

## Data available for sharing

The data presented here were collected through the support of two NIH-funded grants on schizophrenia: (1) Neuromorphometry in Schizophrenia (R01-MH056584), and (2) Conte Center for the Neuroscience of Mental Disorders (P50 MH071616). Through these projects, our group has collected high-resolution sMRI datasets from large cohorts of subjects using the same scanner platform and sequence protocols. We have also collected detailed clinical, cognitive and genetic information from these subjects. In addition, as part of the growing field of Computational Anatomy (CA) (Grenander and Miller, 1998; Csernansky et al., 2004a), our group has developed a suite of tools for mapping brain structures and characterizing schizophrenia-related abnormalities (Haller et al., 1997; Csernansky et al., 2004a; Wang et al., 2007b).

### Subjects

NUSDAST includes de-identified data in compliance with HIPAA^1^ from 451 individuals with schizophrenia, their non-psychotic siblings, comparison subjects and their siblings. Longitudinal data also available on 171 subjects with schizophrenia (m/f = 114/57, age at baseline = 33.8 ± 12.5 years) and 170 controls (m/f = 86/84, age at baseline = 31.4 ± 13.8 years). Within these subjects, 18 subjects with schizophrenia and 30 controls returned for a second follow-up (i.e., 3 time points). The average (SD) follow-up interval was 2.19 (0.82) years for schizophrenia subjects and 2.28 (0.49) years for the controls. See Table 1 below for baseline information.

**Table 1:** **Subject characteristics at baseline**.

	**Schizophrenia subjects**	**Control subjects**	**Schizophrenia siblings**	**Control siblings**
N	171	170	44	66
Age at baseline (year)	33.8 (12.5 [17–63])	31.4 (13.8 [13–67])	N/A	N/A
Gender (male/female)	114/57	86/84	21/23	16/50
Race (Caucasian/African-American/Other)	90/78/3	61/105/2	17/27/0	16/50/0
Global SAPS score	11.1 [12.7 (0–81)]	0.06 [0.3 (0–4)]	0.5 [1.3 (0–7)]	N/A
Global SANS score	9.6 [10.7 (0–62)]	0.04 [1.7 (0–19)]	2.3 [4.8 (0–38)]	N/A

Clinical data includes information based on specific criteria for clinical stability (Rastogi-Cruz and Csernansky, 1997) and clinical rating scales such as the Scale for the Assessment of Positive Symptoms (Andreasen, 1984) and Scale for the Assessment of Negative Symptoms (Andreasen, 1983) (see Table 1 below for baseline information). Domains of psychopathology (i.e., psychotic symptoms, disorganized symptoms, and negative symptoms) (Andreasen et al., 1995) based on raw scales are also included. The reliability and practicality of using these scales in large populations of schizophrenic patients have been demonstrated by Andreasen et al. (1995). Symptom assessments were performed by personnel specially-trained for this purpose. Inter-rater reliability was monitored regularly for all rating scales, and rater training sessions, including the conjoint assessment of difficult cases, were held weekly. In these sessions, a variety of patients were interviewed in a group. Two established raters reached a consensus of item scores after the interview was completed, and then this “gold standard” score was compared with the rest of the group. New raters were trained by first participating in a minimum of six of these sessions. They were allowed to participate in ratings only after they had demonstrated satisfactory agreement with trained personnel.

### MRI data

All MR scans were collected using the same 1.5 T Vision scanner platform (Siemens Medical Systems) at each time point. The Vision scanner had actively shielded gradients and echo-planar capability with very high gradient linearity (<0.4% over a 22-cm diameter spherical volume compared to 2–5% over 22-cm for our other scanners), which yielded anatomical images with virtually no distortion (<0.4% voxel displacement), critical to analyses of neuroanatomical structures. Using the same scanner provided stable longitudinal MR data throughout the entire period of data collection from 1998 to 2006.

Acquisition of all scans was performed at the Mallinckrodt Institute of Radiology at Washington University School of Medicine, where scanner stability (e.g., frequency, receiver gain, transmitter voltage, SNR) and artifacts were regularly monitored. Phantoms of known size were scanned to confirm image dimensions. Further tests and adjustments (shims, gradient calibrations, EPI switch delays, etc.) were made as needed. During each scan session, a small standardization object (i.e., vitamin-E gelcap) was placed on the left side of the forehead for each subject to clearly indicate laterality in the scans. Each scan session included a high-resolution T1 turbo-FLASH scan (Venkatesan and Haacke, 1997) and multiple (2–4) MPRAGE scans. Source MR scan data were in Siemens VISION IMA format and subsequently converted into Analyze™ format using in-house software. Since Analyze-format images may cause confusion with regard to laterality, even though the abovementioned vitamin-E gelcap information may help verify laterality, all Analyze-format images are being converted into NIFTI format and uploaded. The multiple MPRAGE images for each subject are aligned with the first image and averaged to create a low-noise image volume (Buckner et al., 2004). See Table 2 for detailed scan protocol parameters.

**Table 2:** **MR scan parameters**.

**Sequence**	**Protocol parameters**
3D turbo-FLASH	TR = 20 ms, TE = 5.4 ms, flip = 30°, ACQ = 1, 256 × 256 matrix, 1 × 1 mm in-plane resolution, 180 slices, slice thickness 1 mm, 13:30 min scan time
3D MPRAGE (2–4 repeats)	TR = 9.7 ms, TE = 4 ms, flip = 10°, ACQ = 1, 256 × 256 matrix, 1 × 1 mm in-plane resolution, 128 slices, slice thickness 1.25 mm, 5:36 min scan time each

### Neuroimaging meta-data

In our template-based brain mapping applications, we have focused on a network of structures previously implicated in the pathophysiology of schizophrenia (Weinberger et al., 1992; Csernansky and Bardgett, 1998; Goldman-Rakic, 1999). This network included regions with the prefrontal cortex (e.g., middle frontal gyrus—Brodman area 46) (John et al., 2006; Harms et al., 2010), the cingulate gyrus (Qiu et al., 2007; Wang et al., 2007a), and the hippocampus (Wang et al., 2001; Csernansky et al., 2002), the parahippocampal gyrus (Karnik-Henry et al., 2012), as well as the thalamus (Csernansky et al., 2004a; Harms et al., 2007; Smith et al., 2011) and the basal ganglia (Mamah et al., 2007; Wang et al., 2008), which directly or indirectly link these structures via cortical-subcortical connections. We have constructed manual segmentation datasets for all these structures, which can be used for the validation of new computational methods. In addition, we have also used FreeSurfer (Desikan et al., 2006) to generate cortical surface parcellations and measures of cortical regional volume, thickness, and surface area (Cobia et al., 2011).

#### Template data

The templates for the hippocampus and amygdala were generated using a T1-weighted MR scan collected in a healthy subject. The templates for the thalamus and basal ganglia (caudate nucleus, putamen, nucleus accumbens and globus pallidus) were generated using a seven-time averaged T1-weighted MR scan collected in another healthy subject. The segmentations were manually performed in these scans by consensus of experts using atlas guidelines (Duvernoy, 1988, 1991; Mai et al., 1997). Surfaces (byu format) of each structure were generated using the marching cubes algorithm (Lorensen and Cline, 1987; Claudio and Roberto, 1994). The left and right surfaces have corresponding nodes so that analyses of shape asymmetry can be performed.

#### Landmark and surface data

Mapping of the template MR scan occurred in a two-step process. First, it was coarsely aligned to each target scan using landmarks, and then the diffeomorphic map was applied. Surfaces for subcortical structures in the target scans were generated by carrying the template surfaces through these maps (Joshi et al., 1997; Csernansky et al., 2004b).

To facilitate our template-based mapping, global and local (i.e., structure-dependent) neuroanatomical landmarks were placed on the MR images. Landmark-based registration (Joshi et al., 1995) served to adjust the orientation and size for the head (based on global landmarks) and the subcortical structures of interest (based on local landmarks). *Global landmarks*: in each scan, twelve global landmarks were placed following procedures described in Haller et al. (1997): at the points where the anterior and posterior commissures intersected the midsaggittal plane, and at the external boundaries of the cerebrum (anterior, posterior, superior, inferior and lateral). *Local landmarks*: (1) Hippocampus and amygdala were landmarked separately as follows. The most anterior and posterior boundaries of the structure were identified first and a line connecting these points created an anterior/posterior axis. Then in each of five equally distanced slices along this axis, four landmarks were placed at predetermined points in each slice. (2) Thalamus and basal ganglia were landmarked together as follows. The most anterior boundary of the caudate nucleus and the most posterior boundary of the thalamus were identified and a line connecting these points created an anterior/posterior axis. The region between the two points was then divided into five equally distanced slices along this axis and in each slice five landmarks were placed at predetermined places.

#### Freesurfer data

All scans were processed through FreeSurfer Version 3.0.4 (Desikan et al., 2006) pipeline, with careful quality assurance as per FreeSurfer recommendations. All FreeSurfer data, including subcortical segmentation, cortical parcellation and surface, and regional measurement data have been made available.

### Cognitive data

Schizophrenia subjects demonstrate a wide array of cognitive deficits (Gur et al., 2001). Data related to intellect, executive functioning (verbal and visual abstraction), and attention, as well as working and episodic memory are included in this data set. Measures of episodic memory included verbal and visual learning, and also the spontaneous and guided use of memory cues (Jacoby et al., 1993a,b, 2001; Jacoby, 1999). Our assessment of working memory included maintenance and manipulation processes across both verbal and visual modalities, (Braver et al., 1997; Barch et al., 2002). To date, our schizophrenia subjects have demonstrated deficits across all predicted cognitive domains using this battery (Delawalla et al., 2006; Cobia et al., 2011).

At each visit, the subjects were administered a core battery of neuropsychological measures relevant to areas identified in prior studies of cognition in schizophrenia (Nuechterlein et al., 2004). Tasks were grouped into the following four domains:

#### Crystallized intelligence

Scaled scores from the Vocabulary subtest of Wechsler Adult Intelligence Scale [WMS-III; (Wechsler, 1997)].

#### Working memory

Scaled scores based on subtests from the Wechsler Memory Scale—Third Edition WMS-III; (Wechsler, 1997) including Digit Span (total forward and backwards), Spatial Span (total forward and backwards), and Letter-Number Sequencing, and also overall d-prime from the CPT-IP task (Cornblatt et al., 1988).

#### Episodic memory

Included scaled scores from the WMS-III Logical Memory and Family Pictures subtests.

#### Executive function

Included number of novel words generated on phonemic and semantic verbal fluency tasks (Benton, 1976; Benton et al., 1984), time to completion on the Trail Making Test Part B (Reitan and Wolfson, 1985), scaled scores on the WAIS-III Matrix Reasoning subtest, and number of perseverative errors on the Wisconsin Card Sorting Test (Heaton et al., 1993).

Cronbach's alpha (assessed in the standardization set of subjects) was 0.77, 0.78, and 0.70 for working memory, episodic memory, and executive function, respectively, in the individuals with schizophrenia, and 0.76, 0.65, and 0.67, respectively, in the control individuals.

### Genotyping data

Blood for the isolation of DNA was collected from each of the subjects. These samples have been genotyped for a panel of 20 gene polymorphisms selected for their association with schizophrenia or their involvement in neurodevelopment. Examples of these genes include BDNF (rs6265), EGFR (rs10228436), FGF-2 (rs1048201), and IL-3 (rs40401). Morphometric measures (e.g., structural volume) of individuals are compared and contrasted with specific differences (i.e., Single Nucleotide Polymorphisms, or SNPs) in the genes of interest. These differences are qualified by testing whether or not each subject has a particular polymorphism, and then how many copies of that polymorphism they have. A subject can fall into one of three categories: both copies of the gene are polymorphism-free (homozygous), one copy is polymorphism-free whereas the other copy has the polymorphism (heterozygous), or both copies of the gene carry the polymorphism (homozygous).

Presently we have genotyping data on 117 subjects with schizophrenia and 58 controls. DNA samples in additional subjects are being analyzed. All available genotype data will be made available with the scans to users of the database.

### Data dictionary

Along with the data, we provide a data dictionary of terms. In the dictionary, standard descriptions for which ontologies exist are used. We searched following sources for ontology: NeuroLEX (http://neurolex.org/wiki/Main_Page)—a semantic wiki for terms used in Neuroscience, the Neuroscience Information Framework (http://www.neuinfo.org/)—a dynamic resource of Web-based neuroscience data, materials, and tools (NeuroLEX terms are actually published in NIF), and NCI Metathesaurus http://ncimeta.nci.nih.gov/ncimbrowser/)—a biomedical terminology database for translational and basic research. A detail list of these terms is presented in Table 3, and examples include “Socioeconomic Status,” “SAPS,” and “Cognitive Assessment.” For descriptions for which there are no standard ontologies, such as “Working Memory” or “Global Rating of Hallucinations,” we plan to work with NeuroLEX to arrive at standard definitions. The current version of the data dictionary can be downloaded through the data portal website, described below.

**Table 3:** **Table of available ontology for data dictionary terms**.

**Ontology**	**NeuroLex name and ID**	**NIF standard ontology ID**	**NCI methathesaurus ID**
**TERM**			
Gender	Gender assessment birnlex_3026	nif_inv:birnlex_3026	C44177
Race	Race assessment birnlex_3040	nif_inv:birnlex_3040	C17049
Group	Control role birnlex_11017^a^		
Ethnicity	Ethnicity assessment birnlex_3015	nif_inv:birnlex_3015	C16564
Marital status	Marital status assessment birnlex_3031	nif_inv:birnlex_3031	C25188
Type of housing/living arrangement	Living arrangement assessment birnlex_3030	nif_inv:birnlex_3030	C94852
Number of siblings			C102469
Number of children	Offspring cardinality assessment birnlex_3035	nif_inv:birnlex_3035	
Current occupation/job title	Occupation assessment birnlex_3036^b^	nif_inv:birnlex_3036	C25193
Principle occupation	Occupation assessment birnlex_3036^b^	nif_inv:birnlex_3036	C25193
Level of education	Education assessment birnlex_3014^c^	nif_inv:birnlex_3014	
Years of schooling	Education assessment birnlex_3014^c^	nif_inv:birnlex_3014	C17953
Father's level of education	Father's education birnlex_3021^d^	nif_inv:birnlex_3021	
Father's years of schooling	Father's education birnlex_3021^d^	nif_inv:birnlex_3021	
Mother's level of education	Mother's education birnlex_3023^e^	nif_inv:birnlex_3023	
Mother's years of schooling	Mother's education birnlex_3023^e^	nif_inv:birnlex_3023	
Socioeconomic status	Socio-Economic status birnlex_3048	nif_inv:birnlex_3048	C17468
Handedness			(CUI) C0023114
Edinburg handedness assessment	Edinburg handedness assessment birnlex_3013	nif_inv:birnlex_3013	
SAPS	Scale for the Assessment of Positive Symptoms birnlex_3045^f^	nif_inv:birnlex_3045	
SANS	Scale for the Assessment of Negative Symptoms birnlex_3041^g^	nif_inv:birnlex_3041	
Cognitive assessment	birnlex_2021	nif_inv:birnlex_2021	C0870300

a Currently a proxy class to be replaced by its OBI (The Ontology for Biomedical Investigations) equivalent.

b NeuroLex Occupation assessment includes work specialties as defined by duties and required skills as well as principal activity that a person does to earn money.

c NeuroLex Educational assessment includes level of education and years of schooling.

d NeuroLex Father's education assessment includes father's level of education and father's years of schooling.

e NeuroLex Mother's education assessment includes mother's level of education and mother's years of schooling.

f NeuroLex SAPS describes a type of assessment without any defined attributes.

g NeuroLex SANS describes a type of assessment without any defined attributes.

## Data sharing mechanism

### Data sharing architecture: XNAT and XNAT central

The collected MR datasets along with detailed clinical and cognitive information are archived using the eXtensible Neuroimaging Archive Toolkit (XNAT), an open source data management and productivity platform for biomedical imaging research. XNAT was developed by Neuroinformatics Research Group (NRG) at Washington University in St. Louis and the BIRN (Marcus et al., 2007a,b). It is widely used across the world and is a core component of the emerging NIH-backed biomedical informatics backbone, including the Biomedical Informatics Research Network (BIRN) and National Alliance for Medical Image Computing (NA-MIC). XNAT includes a secure database backend and a rich web-based user interface.

XNAT Central (http://central.xnat.org/) is a public access repository for neuroimaging and related data operated by the NRG. XNAT Central is built on the XNAT data management platform. XNAT Central includes a number of secure tools for storing and accessing images including a DICOM server, a web services API, and a user friendly website. Non-imaging data (e.g., demographics, clinical measures, derived measures) can be uploaded via spreadsheets or online forms. The website allows users to easily search for data across the various imaging and non-imaging measures, to visualize raw and processed images, and to download selected datasets.

### XNAT custom schemas

XNAT relies on Extensible Markup Language (XML) schema documents to define the type of data that can be stored in the system. XML is the standard language for defining open and extensible data formats. XML format provides a number of benefits for data organization: it provides a way to uniformly describe data and data structure, it makes data available to consistent and efficient programmatic manipulation, reuse, transmission and storage, and it simplifies data conversion to other formats. XNAT comes with a set of XML schemas that describe common data associated with neuroimaging studies. XNAT also allows for the extension of these schemas as well as the creation of custom schemas. NUSDAST contains all three types: common, extended, and custom schemas. The extended and custom schemas include: subject registration data, extended demographic and relationship information, psychopathology measures based on SAPS and SANS, cognitive data and SNP data.

#### Custom subject registration schema

The registration information describes each subject's diagnostic category and longitudinal follow-up status and timing. We extended the standard XNAT base schema type subjectAssessorData to define a complex EncounterLog type schema. It is now a standard XNAT base schema available for any users of XNAT Central interested in preserving subject continuity information related to a study. The custom EncounterLog schema describes data listed in Supplemental Table ST1 and Supplemental Figure SF1.

#### Custom demographics and relationship schema

The standard XNAT base schema type demographicData was extended to define a complex nundaDemographicData type schema. The standard XNAT base demographic type describes Date of Birth, Year of Birth, Age, Gender, Handedness, Socioeconomic status, Education, Education description, Race, Ethnicity, Weight, Height, Gestational age, Post menstrual age, Birth weight. The extended nundaDemographicData and relationship schema describes additional data listed in Supplemental Table ST2 and Supplemental Figure SF2.

#### Custom psychopathology schema

We extended the standard XNAT base schema type subjectAssessorData to define a complex symptomsSAPSSANS type schema. The custom symptomsSAPSSANS schema describes data listed in Supplemental Supplemental Table ST3 and Supplemental Figure SF3.

#### Custom cognition schema

We extended the standard XNAT base schema type subjectAssessorData to define a complex symptomsNeurocog type schema. The custom symptomsNeurocog schema describes data listed in Supplemental Table ST4 and Supplemental Figure SF4.

#### Custom genetics schema

We created the genetic schema extension to accommodate schizophrenia-related genetic information. The custom genetics schema describes data listed in Supplemental Table ST5 and Supplemental Figure SF5.

### Data standardization for sharing within XNAT

In data sharing, it is essential for different projects with the same/similar data elements to use the same/similar schema, not different schemas, to describe the data. Toward this end, we first recognize that several of our assessments used industry-standard instruments that many other groups use, particularly in schizophrenia research. These instruments include the SAPS/SANS assessments for psychopathology and items of cognitive battery (e.g., WMS-III above). Custom schemas for these assessments have now been made standard within XNAT Central for ready use by other projects.

On the other hand, other data were obtained by non-standard assessment instruments but are nonetheless valuable for other projects. These data include the SNP (e.g., BDNF-rs6265 allelle) and sibling relationships data (i.e., which two subjects are siblings and whether the sibling is a brother or sister). Custom schemas for these assessments are available in XNAT Central for ready adaptation by other projects.

Besides creating these new, custom schemas, we also took existing, XNAT standard schemas and extended to accommodate our data. These include the demographics and relationship schema. This extended schema is applicable to any XNAT Central project that preserves subject continuity information. We therefore have made it a standard base schema in XNAT Central.

## Data translation and upload into XNAT central

**Figure 1:**
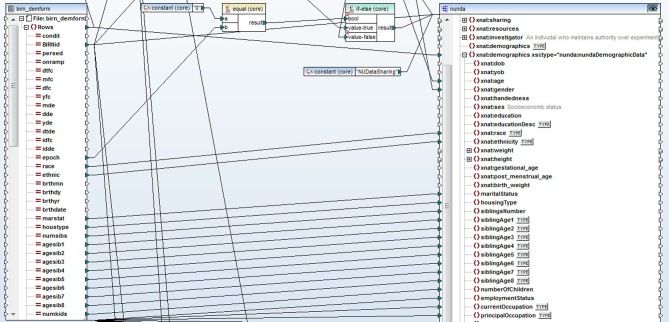
**A snapshot of a segment of the Altova MapForce mapping project.** Data is represented on the left side of the screen, XNAT XML schema document is on the right. Data transformation functions and visual mapping tool define the rules for the transformation.

The non-imaging data were stored in a number of different source architecture types such as excel spreadsheets, SAS and SPSS data files. We used Altova MapForce software (Altova GmBH, 2012) to convert the data into XML format. Altova MapForce provides the ability to create complex mappings between data in a variety of formats and the documents that define structure and relationships of data. In this case, we used Altova MapForce to transform the data into XML documents compliant with the XNAT (see XNAT Central below) XML schema extensions that we designed for each type of data. As a result, MapForce generated a set of XML files that contained demographic, clinical, symptomatic, registration and cognitive data. See Figure 1 for a snapshot of the Altova MapForce mapping project segment, demonstrating data transformation functions and the visual mapping tool.

Our XNAT Central repository is entitled “NU Schizophrenia Data and Software Tool Federation using BIRN Infrastructure (NUSDAST).” Uploading the XML data files was facilitated by the use of Python library PyXNAT (http://packages.python.org/pyxnat/) (Schwartz, 2011; Schwartz et al., 2012), which is built on top of XNAT's REST API. PyXNAT provides data management and the ability to connect with other existing neuroimaging software, such as FreeSurfer, to the data in the repository, as well as the ability to distribute data to high performance computing clusters for performing CPU-intensive data analysis tasks. Non-XML data (such as surfaces and landmarks) were uploaded via cURL (http://curl.haxx.se/), also over the REST API. We are continuing to upload data as new schema extensions on the XNAT Central repository are being created and implemented.

## Data access

### XNAT central

**Figure 2:**
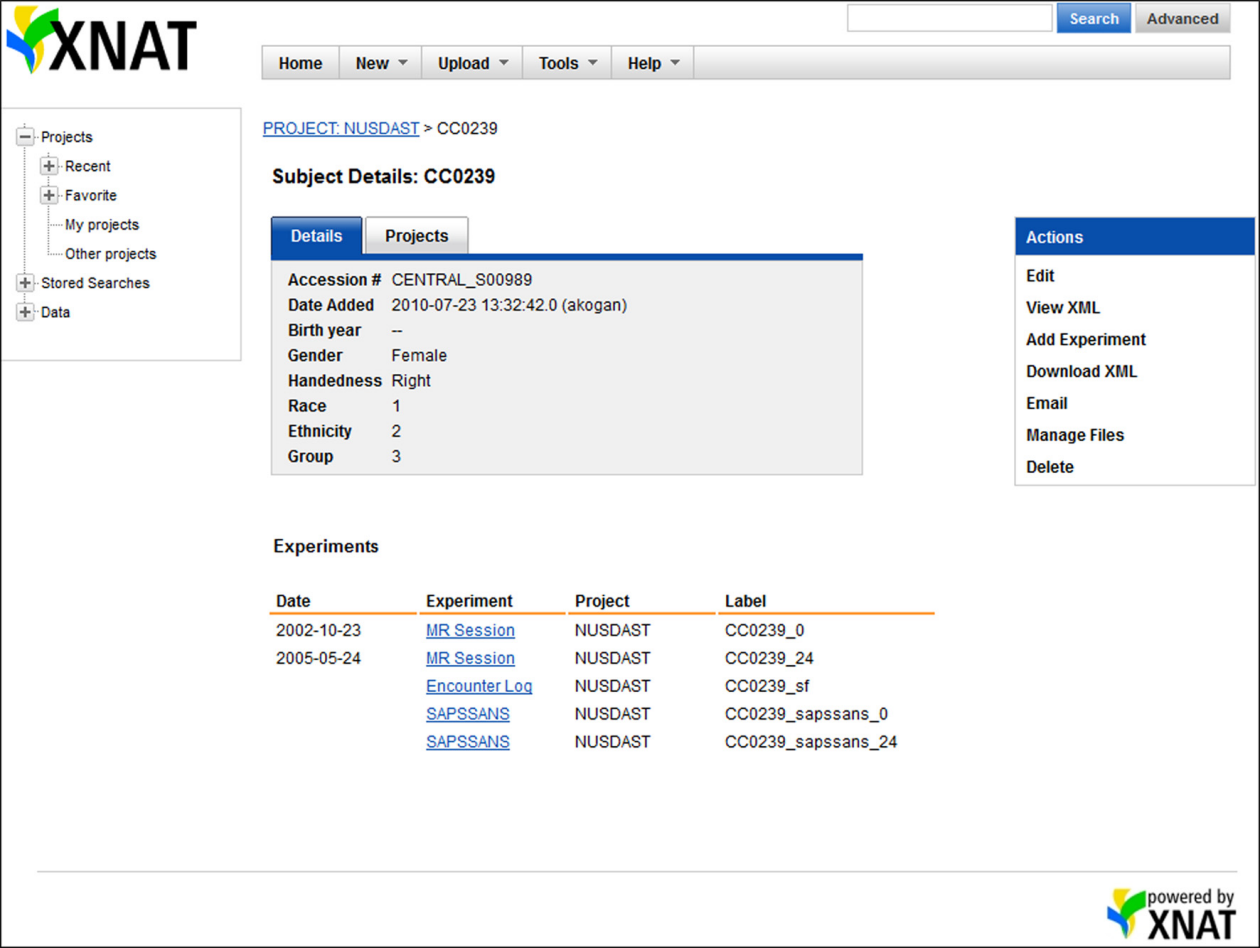
**Screenshot of XNAT Central data.** On the XNAT Central website, all data related to a particular subject, e.g., MR sessions, study registration, symptoms, and cognitive battery, are organized under the subject.

The project “NU Schizophrenia Data and Software Tool Federation using BIRN Infrastructure (NUSDAST)” is hosted on XNAT Central (http://tinyurl.com/av9h7jm) ^2^Within the NUSDAST project on the XNAT Central website, data are organized by subjects following the XNAT architecture: study registration, longitudinal epochs of MR sessions, symptoms, and cognitive battery (Figure 2). Within each epoch's MR session, scans and associated segmentations, surfaces, landmarks, and other data are listed. User download is accomplished via the Download action or the Manage Files action displayed on the XNAT web page. Data can also be retrieved via the XNAT REST API.

### Web-based domain-specific portal built interfacing XNAT

We have created a web-based data portal (http://niacal.northwestern.edu/xnat_queries/NUSDAST) that acts as an interface between the user and the XNAT Central database. The data portal was designed to accommodate NUSDAST-specific queries as well as queries that will suit the user's needs. While XNAT Central has an excellent search query interface, it is lacking certain important features that could facilitate domain-specific data query and access. One important point is that without registration at XNAT Central, one does not know whether NUSDAST contains their desired data. A very useful and important feature of the data portal is that anyone to obtain a summary count of their search criteria before registering at XNAT Central. For example, a search for “female” and “2 or more” MR sessions (i.e., longitudinal imaging data only) would return with “*We have 73 subjects (164 MR imaging sessions) matching the parameters you specified*.” The user then can decide to register with XNAT Central and obtain detailed data.

Another important feature of the portal is our ability to provide NUSDAST-specific information. For example, in an XNAT Central advanced search, the user would have to specify before-hand that he or she would like to have cognitive data even though it is not part of the search criteria, for otherwise cognitive data would not be included as part of the data download after the search is complete. Our portal can be customized to give the user options of selecting what data they would like to have to download, after the subjects satisfying the search criteria are returned. For example, a user searching on age and imaging type parameters may want to download cognitive data for the subjects fitting search criteria.

**Figure 3:**
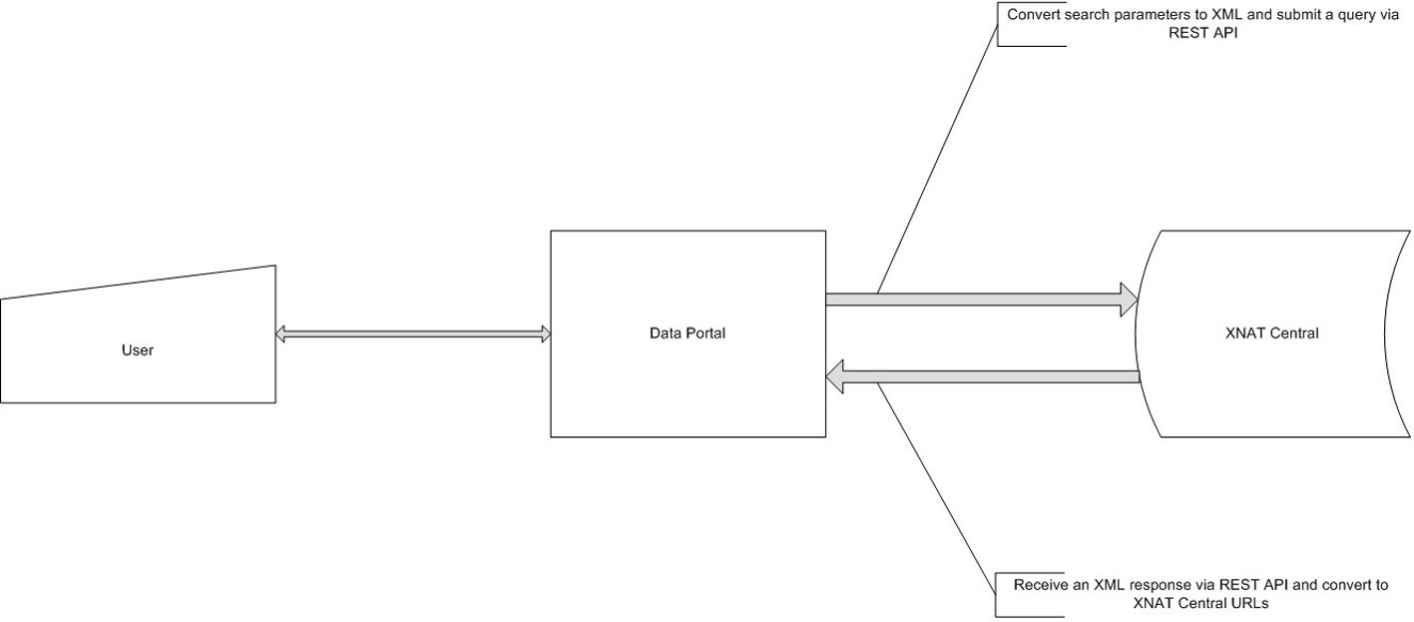
**Data Portal dataflow diagram.** Data Portal creates a request, based on user's criteria and queries XNAT Central server. After the receipt of a response it is presented to a user for subsequent review and data retrieval.

**Figure 4:**
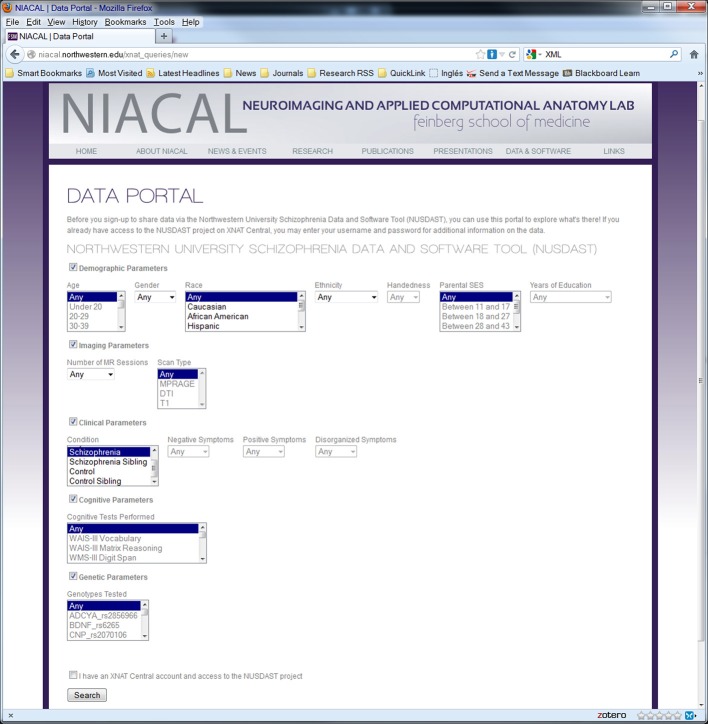
**Screenshot of web data portal.** Data Portal is used to search the collected dataset directly at the repository. Search results can be used to download data.

The data portal was developed as part of a Ruby on Rails (http://rubyonrails.org) application and uses the HTTParty gem (http://httparty.rubyforge.org/) to communicate with the instance of XNAT via REST API. The portal presents options to specify search parameters for demographic, psychopathological, cognitive, genetic, and neuroimaging information that can be used to narrow the dataset which the user is looking to acquire. The application then sends a request to XNAT, in XML format, based on the search parameters specified and receives a list of data, also in XML format, that satisfies the specified criteria. The results are parsed, formatted and displayed on the data portal website along with instructions for downloading this data from XNAT Central. See Figures 3, 4 for a dataflow diagram and a screenshot of the Data Portal. The user can also follow the XNAT Central link to browse and download NUSDAST data directly from XNAT Central. A download feature is available for data to be downloaded from the Data Portal (instead of directly interacting with XNAT Central), achieving a “one-click” capability.

## Neuroimaging analysis tools

### Caworks

**Figure 5:**
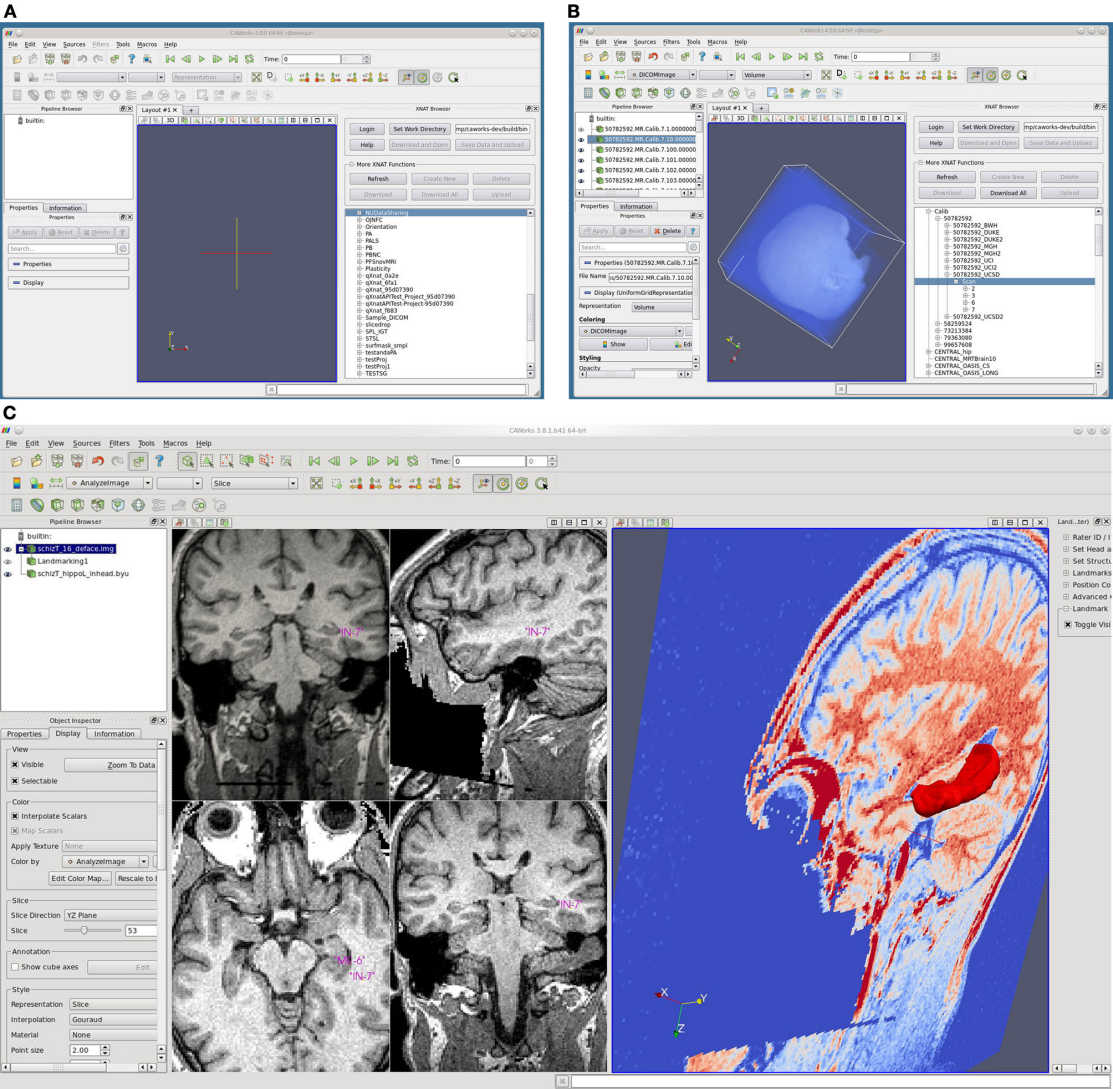
**Screenshots of CAWorks. (A)** CAWorks interface to MR data on XNAT Central [**(B)** NUSDAST is shown in CAWorks GUI as a folder when connected to XNAT Central]. **(C)** Hippocampal landmarks and surface viewed in CAWorks.

The software application Computational Anatomy Works (CAWorks, Figure 5), available at http://www.cis.jhu.edu/software/caworks/index.php, was developed to support computational anatomy and shape analysis. CAWorks works seamlessly with the NUSDAST image, landmark and surface data and the Large Deformation Diffeomorphic Metric Mapping (LDDMM) mapping engine (Beg et al., 2005). The capabilities of CAWorks include:

1. Image and Shape Analysis plugin modules, such as Large Deformation Diffeomorphic Metric Mapping (LDDMM).
2. Interactive landmark placement to create segmentation (mask) of desired region of interest and specialized landmark placement plugins for the hippocampus, amygdala, and entorhinal cortex. After landmarking is completed, CAWorks facilitates submission for automated segmentation processing (e.g., LDDMM).
3. Browser plugin module for XNAT, enabling the retrieval of medical image data from XNAT for image and shape analysis and the storage of results in XNAT.
4. Quadra Planar view visualization.
5. Support for multiple Medical Imaging data formats, such as Nifti, Analyze, Freesurfer, DICOM, and landmark data.

CAWorks provides a GUI interface to any specified XNAT repository (including XNAT Central) such that the XNAT data appears as folders in a hierarchical structure (Figure 5 panels A and B). When data is selected, it is cached locally and opened in the visualization window. The save option uploads and updates the XNAT repository. CAWorks gives researchers a more productive and effective experience in the analysis and visualization of data stored within XNAT. The direct interface with XNAT provides an intuitive hierarchical organizational structure which users may traverse and explore data. The user simply clicks on the data to be visualized and it appears in the interactive window. This saves the researcher the extra steps of uploading, downloading, and organizing the data temporarily on the file system.

## Discussion

In this paper, we described the development and deployment of a usable combination of schizophrenia-related dataset, tools for storing, sharing, support for computational anatomy and a data-mining portal, the NUSDAST.

Concerning data sharing, this resource built and extended upon existing, standard schemas available for data sharing on XNAT Central (http://central.xnat.org/). Specifically, we developed additional schemas for storing demographic, cognitive, genetic and clinical meta data in XNAT. These additions create the opportunity to consistently expand and share schizophrenia research-related data^3^. We have significantly improved the way scientists are able to mine our dataset by creating a data portal for searching and downloading our data along with the accompanying longitudinal data.

Our well-described and comprehensive data on the normal controls and their siblings are valuable beyond the schizophrenia research community. For example, The SNP data include ones that are related to neurodevelopment (e.g., BDNF), embryonic development and tissue repair (e.g., FGF-2), and immune response/inflammation (e.g., EGFR, IL-3). Mutations of many of these SNPs have been found to be related to cancer and neuropsychiatric disorders such as depression, anxiety and Alzheimer disease. Therefore, with the accompanying imaging and cognitive data, our control subjects data can be of wider utility beyond schizophrenia research.

Concerning data exploration, this source offers CAWorks, which extends ParaView, an open source, multi-platform, freely available program for parallel, interactive, scientific visualization. An important visualization tool for TeraGrid researchers, its client-server architecture facilitates remote visualization of datasets and the generation of level of detail (LOD) models that maintain interactive frame rates for large datasets. CAWorks is being used by consortium and center projects such as the Biomarkers for Older Controls At Risk for Dementia (BIOCARD), F.M. Kirby Research Center for Functional Brain Imaging, and BIRN projects. Additional functionality is being developed for storing and retrieving XNAT data as new analysis tools are added to CAWorks.

The schizophrenia research community has invested substantial resources in order to collect, manage and share increasingly larger datasets including neuroimaging data. The exploration of large, multi-modal, datasets has indeed improved our understanding of relationships among abnormalities of brain circuitry, brain function and genetic variability in schizophrenia (Kim et al., 2009, 2010; Allen et al., 2011).

Numerous data sharing initiatives were undertaken in order to create publicly accessible neuroimaging data collections, such as: FBIRN, MCIC, BSNIP, fMRI DataCenter (fMRIDC), the Open Access Series of Imaging Studies (OASIS). One of the main obstacles to the open-access sharing of research data is the lack of local organization and standard descriptions (Poline et al., 2012). Different resources usually organize data in many different formats, which leads to difficulties in sharing and analyzing data. For example, the acquisition of a dataset from any given source will most likely require some programming work in order to make the dataset suitable for processing by an already utilized suite of tools. The concept of “one-click share” can only come true when appropriate standards are adopted and followed by the research community. The processes that will help establish standardized procedures include proliferation of open-source data sharing and of storage mechanisms. These systems have to respond to the current needs of researchers, to be comprehensive enough (or extensible) to accommodate different types of research and to provide data-mining and common data processing functionality.

It is our hope that this effort will help to overcome some of the commonly recognized technical barriers in advancing neuroimaging research (Poline et al., 2012) by creating a research-ready dataset that meaningfully combines neuroimaging data with other relevant information. Currently, more data are being made available, such as fMRI and genome-wide scan (GWS) data. We are also beginning to expand the scope of schizophrenia neuroimaging data sharing by linking NUSDAST with FBIRN and MCIC through the development of SchizConnect, which will be a data mediation and integration platform that establishes a true federation of disparate, heterogeneous neuroimaging-related databases.

## Conflict of interest statement

The authors declare that the research was conducted in the absence of any commercial or financial relationships that could be construed as a potential conflict of interest.

## Footnotes

^1^De-identification consisted of stripping HIPAA-mandated identifiable information in research data (such as name, initials, and phone numbers, etc.). Procedures to further anonymize imaging data such as defacing were not performed in order to share the same imaging data that we used in our publications so that others can replicate our findings using their own algorithms if they so desire.

^2^Full URL: https://central.xnat.org/app/template/XDATScreen_report_xnat_projectData.vm/search_element/xnat:projectData/search_field/xnat:projectData.ID/search_value/NUDataSharing

^3^Extended and new schemas created by NUSDAST can be used by other existing or future projects to describe their data. A benefit of sharing data through XNAT Central, rather than through an independent XNAT system, is that multiple projects can easily use the same schema representations and user interface components to represent common data elements. Users can then query and mine XNAT Central to locate data across the multiple projects using the shared schema.

## Supplementary material

Supplemental Figure SF1 **Custom EncounterLog schema**.

Supplemental Figure SF2 **Extended nundaDemographicData and relationship schema**.

Supplemental Figure SF3 **Custom symptomsSAPSSANS schema**.

Supplemental Figure SF4 **Custom symptomsNeurocog schema**.

Supplemental Figure SF5 **Custom genetics schema**.

Supplemental Table ST1 **Custom EncounterLog schema**.

Supplemental Table ST2 **Extended nundaDemographicData and relationship schema**.

Supplemental Table ST3 **Custom symptomsSAPSSANS schema**.

Supplemental Table ST4 **Custom symptomsNeurocog schema**.

Supplemental Table ST5 **Custom genetics schema**.
