# Supplementary material for: Northwestern University Schizophrenia Data and Software Tool (NUSDAST)
Source: Front Neuroinform. 2013 Nov 7;7:25. doi: 10.3389/fninf.2013.00025 (PMC3819522; doi:10.3389/fninf.2013.00025)

Supplemental Table ST1. Custom EncounterLog schema.

**Data Field**	**Description**
date	Visit date
time	Visit time
encounter_type	Type of encounter
project_timepoint	Months from baseline
subject_status	Subject status at timepoint encounter
comment	Comment at timepoint encounter

Supplemental Table ST2. Extended nundaDemographicData and relationship schema.

Data Field	Description
maritalStatus	Marital status
housingType	Type of housing
siblingsNumber	Number of siblings
siblingAge1	Age of sibling 1
siblingAge2	Age of sibling 2
siblingAge3	Age of sibling 3
siblingAge4	Age of sibling 4
siblingAge5	Age of sibling 5
siblingAge6	Age of sibling 6
siblingAge7	Age of sibling 7
siblingAge8	Age of sibling 8
NumberOfChildren	Number of children
employmentStatus	Employment status
currentOccupation	Current occupation
principalOccupation	Principal occupation
educationLevel	Highest degree earned
yearsOfSchooling	Number of years attended school
attendSpecialClasses	Attended special education classes?
specialClass	Type of special education class
educationLevelFather	Father’s highest degree earned
schoolingLevelFather	Number of years father attended school
educationLevelMother	Mother’s highest degree earned
schoolingLevelMother	Number of years mother attended school
sesDesc	
handednessExam/writing	Preferred hand for writing
handednessExam/throwing	Preferred hand for throwing
handednessExam/scissors	Preferred hand for scissors
handednessExam/toothbrush	Preferred hand for toothbrush
handednessExam/knife	Preferred hand for knife
handednessExam/spoon	Preferred hand for spoon
handednessExam/broom	Preferred hand for broom
handednessExam/match	Preferred hand for match
handednessExam/box	Preferred hand for box
handednessExam/footkick	Preferred hand for foot kick
handednessExam/eye	Preferred sighting eye
relationship/otherID	Subject ID in the study
relationship/relationshipType	Relationship to subject

Supplemental Table ST3. Custom symptomsSAPSSANS schema.

Data Field	Description
saps1	Auditory Hallucination
saps2	Voices Commenting
saps3	Voices Conversing
saps4	Somatic or Tactile Hallucinations
saps5	Olfactory Hallucinations
saps6	Visual Hallucinations
saps7	Global Rating of Hallucinations
saps8	Persecutory Hallucinations
saps9	Delusions of Jealousy
saps10	Delusions of Guilt or Sin
saps11	Grandiose Delusions
saps12	Religious Delusions
saps13	Somatic Delusions
saps14	Delusions of Reference
saps15	Delusions of Being Controlled
saps16	Delusions of Mind Reading
saps17	Thought Broadcasting
saps18	Thought Insertion
saps19	Though Withdrawal
saps20	Global Rating of Delusions
saps21	Clothing and Appearance
saps22	Social and Sexual Behavior
saps23	Aggressive and Agitated Behavior
saps24	Repetitive or Stereotyped Behavior
saps25	Global Rating of Bizarre Behavior
saps26	Derailment
saps27	Tangentiality
saps28	Incoherence
saps29	Illogicality
saps30	Circumstantiality
saps31	Pressure of Speech
saps32	Distractible Speech
saps33	Clanging
saps34	Global Rating of Positive Thought

Data Field	Description
sans1	Unchanging Facial Expressions
sans2	Decreased Spontaneous Movement
sans3	Paucity of Expressive Gestures
sans4	Poor Eye Contact
sans5	Affective Nonresponsivity
sans6	Inappropriate Affect
sans7	Lack of Vocal Infections
sans8	Global Rating of Affective Flattening
sans9	Poverty of Speech
sans10	Poverty of Content of Speech
sans11	Blocking
sans12	Increased Latency of Response
sans13	Global Rating of Alogia
sans14	Grooming and Hygiene
sans15	Inpersistance at Work or School
sans16	Physical Anergia
sans17	Global Rating of Avolution Apathy
sans18	Recreational Interests and Activities
sans19	Sexual Activity
sans20	Ability to feel Intimacy and Closeness
sans21	Relationships with Friends and Peers
sans22	Global Rating of Anhedonia-Asociality
sans23	Social Inattentiveness
sans24	Inattentiveness During Mental Status Testing
sans25	Global Rating of Attention

Supplemental Table ST4. Custom symptomsNeurocog schema.

**Data Field**	**Description**
dfbv	Days from baseline value
yfbv	Years from baseline value
z_iq_2grp	*Crystallized IQ* z-score of cognitive domain score based on 2-group calculations
z_wm_2grp	*Working Memory* z-score of cognitive domain score based on 2-group calculations
z_em_2grp	*Episodic Memory* z-score of cognitive domain score based on 2-group calculations
z_ra_2grp	*Reasoning Ability* z-score of cognitive domain score based on 2-group calculations
z_pos_2grp	*Positive Symptoms* z-score of psychopathology based on 2-group calculations
z_neg_2grp	*Negative Symptoms* z-score of psychopathology based on 2-group calculations
z_dis_2grp	*Disorganized Thought* z-score of psychopathology based on 2-group calculations
z_iq_4grp	*Crystallized IQ* z-score of cognitive domain score based on 4-group calculations
z_attn_4grp	*Attention* z-score of cognitive domain score based on 2-group calculations
z_wm_4grp	*Working Memory* z-score of cognitive domain score based on 4-group calculations
z_em_4grp	*Episodic Memory* z-score of cognitive domain score based on 4-group calculations
z_ra_4grp	*Reasoning Ability* z-score of cognitive domain score based on 4-group calculations
z_pos_2grp	*Positive Symptoms* z-score of psychopathology based on 4-group calculations
z_neg_2grp	*Negative Symptoms* z-score of psychopathology based on 4-group calculations
z_dis_2grp	*Disorganized Thought* z-score of psychopathology based on 4-group calculations

Supplemental Table ST5. Custom genetics schema.

**Data Field**	**Description**
name	Gene name
genotype	Genotype
minorpos	Minor Positive Allele
heteropos	Heterozygous Positive Allele

Supplemental Figure SF1.Custom EncounterLog schema.

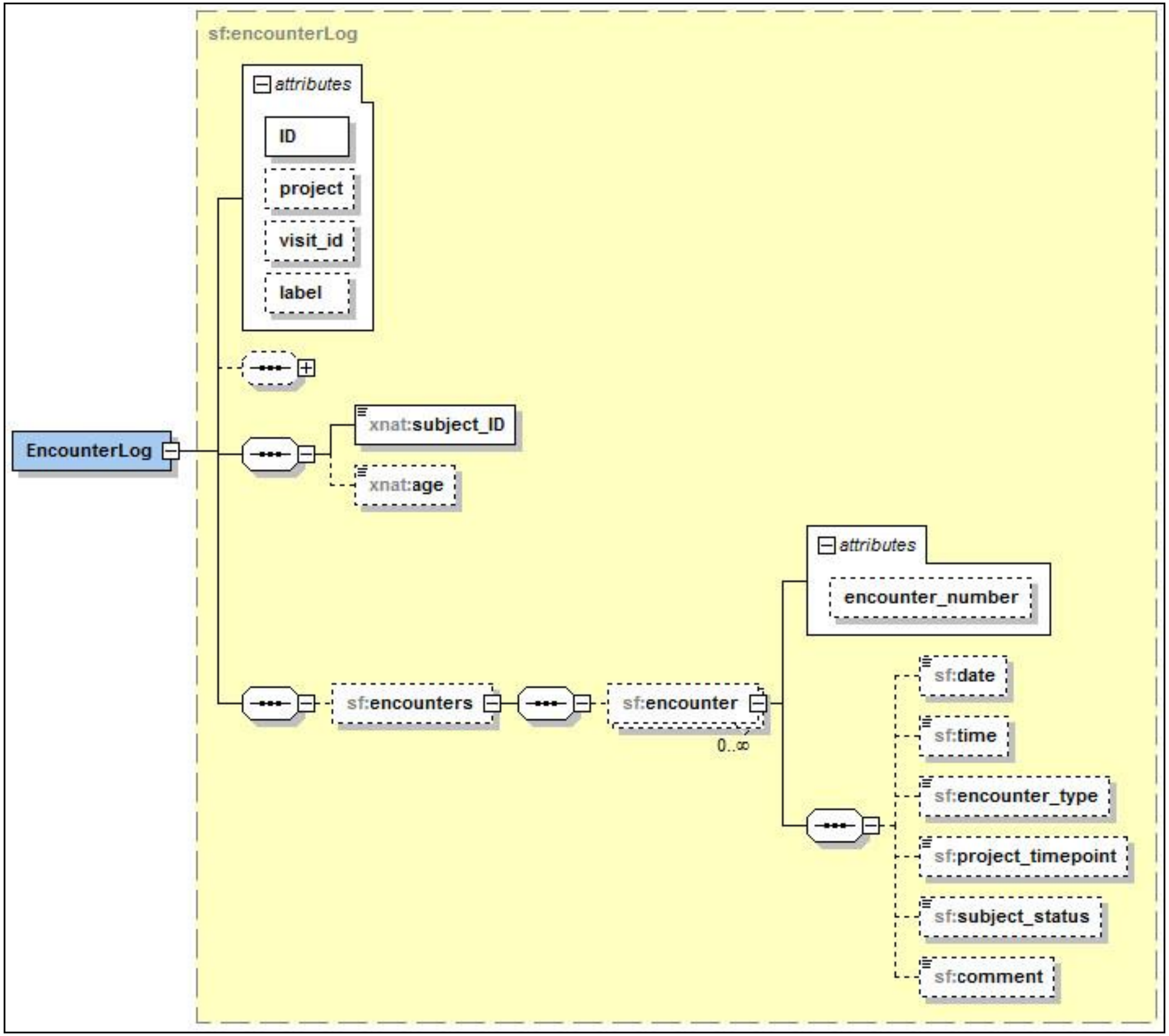

Supplemental Figure SF2. Extended nundaDemographicData and relationship schema.

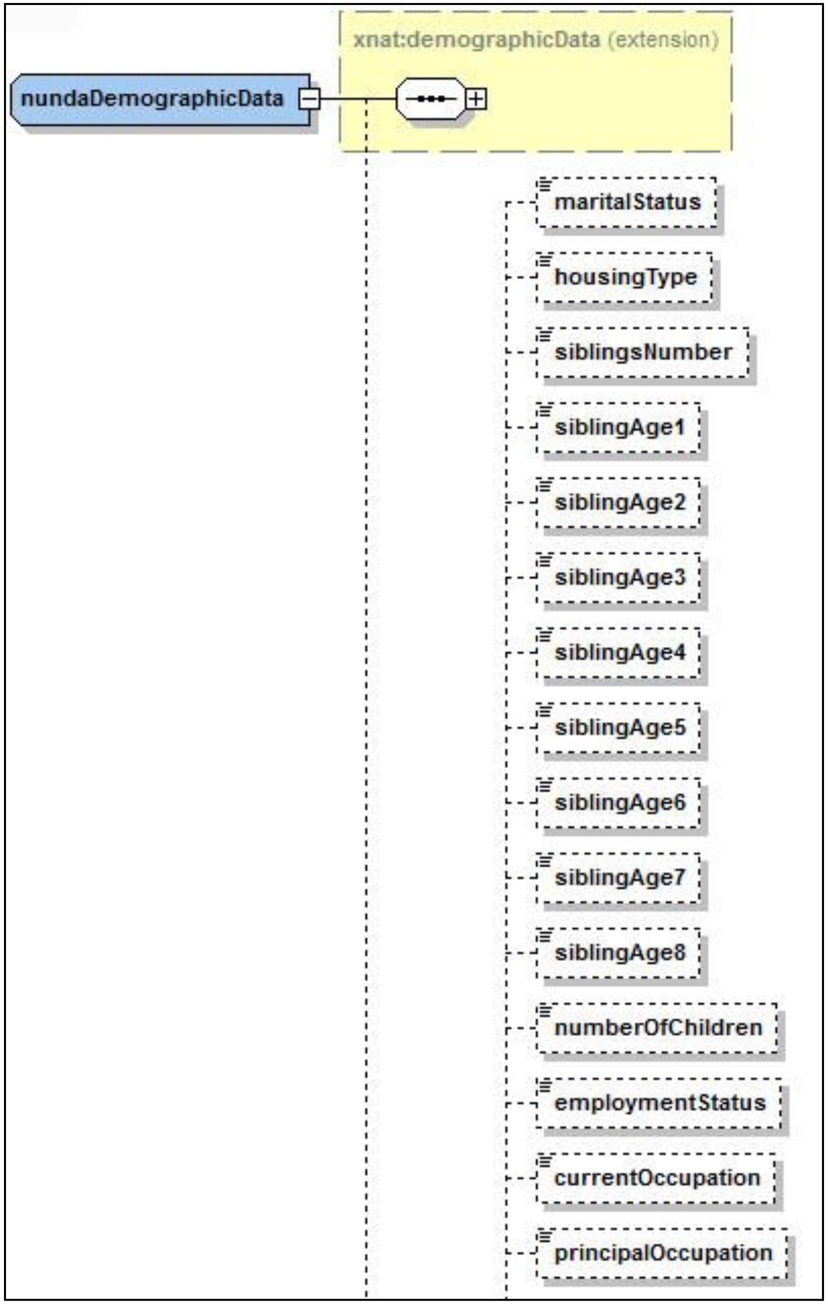

Supplemental Figure SF3. Custom symptomsSAPSSANS schema.

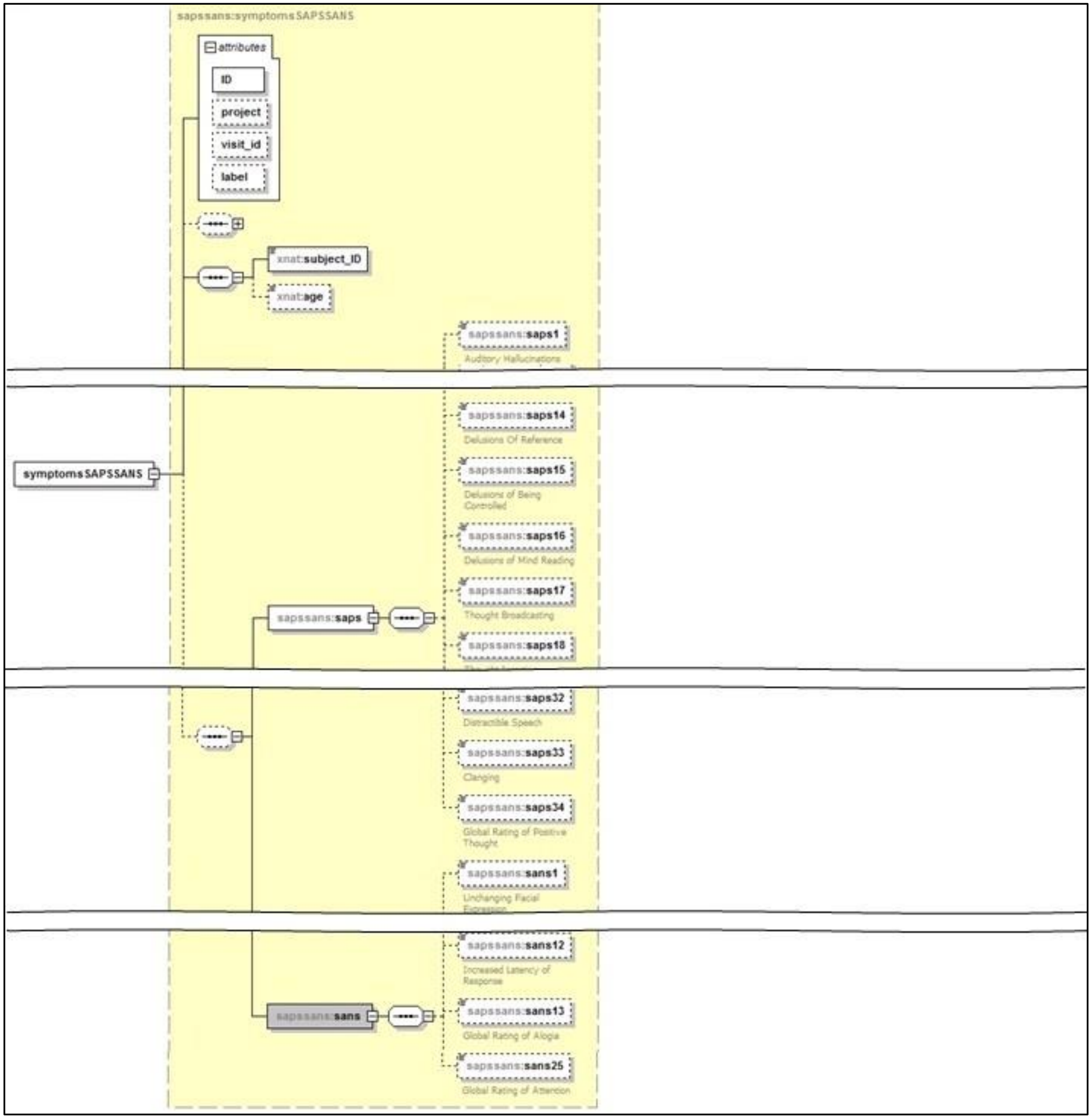

Supplemental Figure SF4. Custom symptomsNeurocog schema.

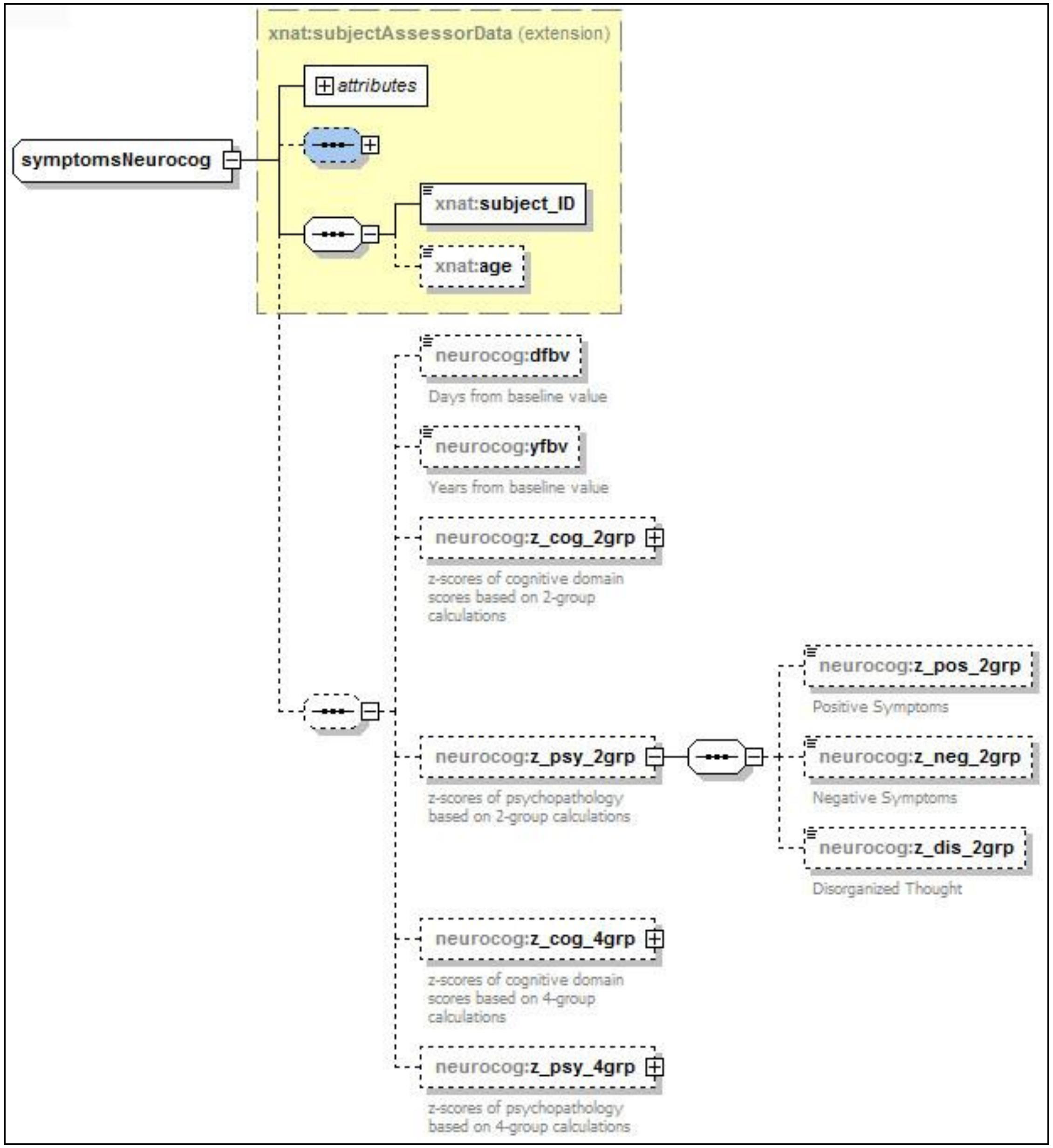

Supplemental Figure SF5. Custom genetics schema.

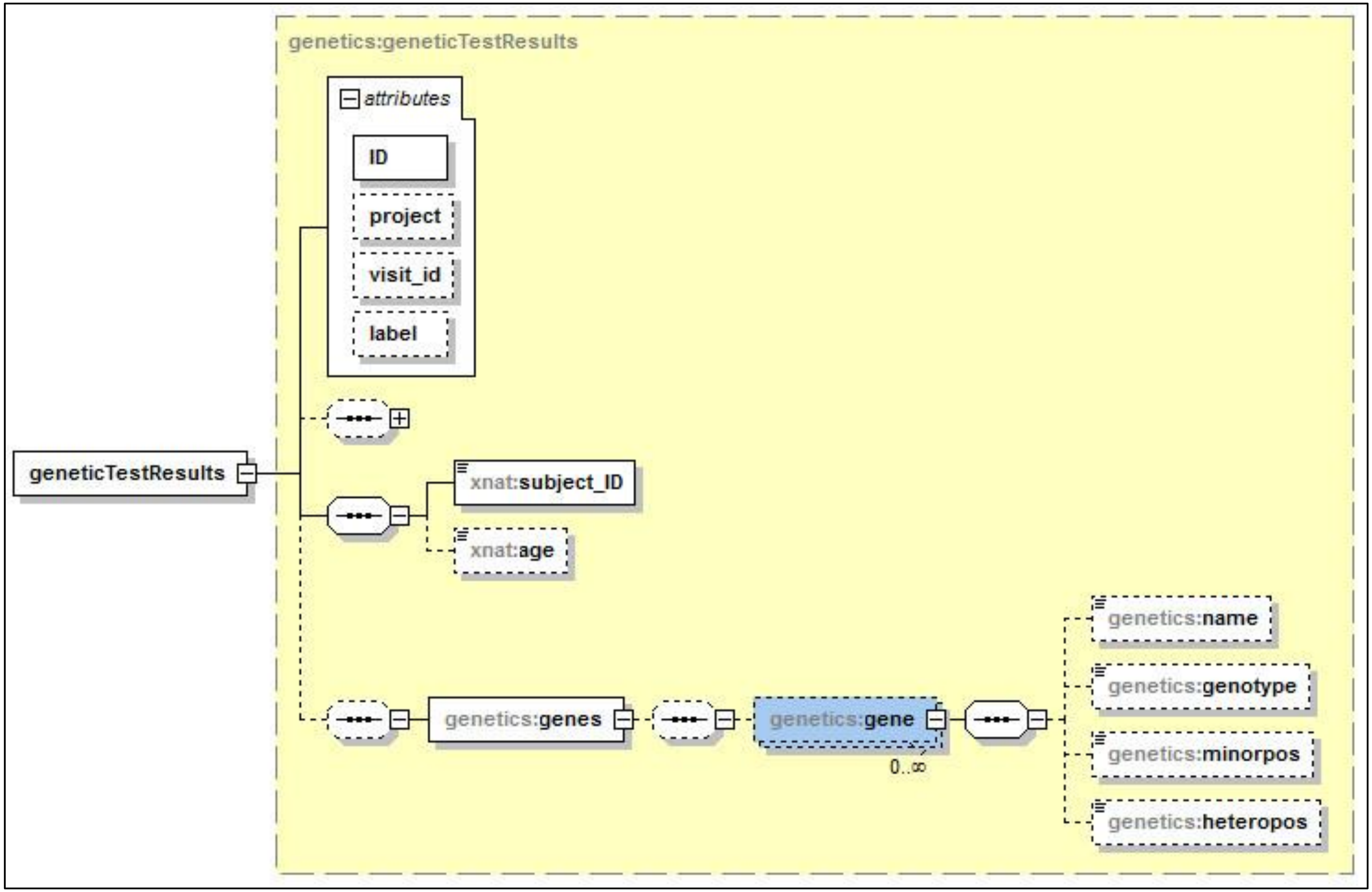

Supplement: Supplemental Figure SF1 — Custom EncounterLog schema. [file DataSheet1.PDF]
